# Relationship between coronary function testing and migraine: results from the Women’s Ischemia Syndrome Evaluation-Coronary Vascular Dysfunction project

**DOI:** 10.20517/2574-1209.2021.55

**Published:** 2021-08-01

**Authors:** Jessica Siak, Chrisandra L. Shufelt, Galen Cook-Wiens, Bruce Samuels, John W. Petersen, R. David Anderson, Eileen M. Handberg, Carl J. Pepine, C. Noel Bairey Merz, Janet Wei

**Affiliations:** 1 Barbra Streisand Women’s Heart Center, Smidt Heart Institute, Cedars-Sinai Medical Center, Los Angeles, CA 90048, USA.; 2 Biostatistics and Bioinformatics Core, Cedars-Sinai Medical Center, Los Angeles, CA 90048, USA.; 3 Division of Cardiovascular Medicine, University of Florida, Gainesville, FL 32608, USA.

**Keywords:** Women, coronary microvascular dysfunction, migraines, angina

## Abstract

**Aim:**

To determine the relationship between coronary vascular dysfunction and history of migraines in women with suspected ischemia and no obstructive coronary arteries (INOCA).

**Methods:**

In the Women’s Ischemia Syndrome Evaluation-Coronary Vascular Dysfunction study, 402 women with suspected INOCA answered baseline angina questionnaires, including the Seattle Angina Questionnaire (SAQ). Coronary function testing (CFT) performed in a subgroup of 252 women evaluated for nonendothelial and endothelial-dependent coronary vascular function. Wilcoxon rank sum test, *t*-test, and linear regression models were performed.

**Results:**

Of the 252 women who underwent CFT, 126 (50%) women reported migraine history. Compared to women who reported no migraines, women with migraines were younger and more were premenopausal. They had more angina at rest, with strong emotions, and hot/cold temperatures, as well as angina that wakes them from sleep (*P* < 0.05 for all). Women with migraines also scored worse on SAQ angina frequency and quality of life *P* < 0.01 for both). There was no difference in prevalence of coronary vascular dysfunction in the two groups. In addition, linear regression models demonstrated no significant age-adjusted differences in absolute CFT variables.

**Conclusion:**

Among women with suspected INOCA, migraine history is prevalent and women with migraines have worse angina compared to those without migraines. Coronary vascular dysfunction diagnosed by CFT does not appear to relate to migraine history.

## INTRODUCTION

Migraine headaches are associated with an increased risk of cardiovascular adverse outcomes, with accumulating evidence suggesting more elevated risk among women^[1]^. The Women’s Ischemia Syndrome Evaluation (WISE) found that, among women with signs and symptoms of ischemic heart disease, those with history of migraine headaches had an 83% excess major cardiovascular event risk at long-term follow-up, adjusted for traditional cardiovascular risk factors and coronary artery disease severity^[2]^. Endothelial function and vasomotor reactivity have been hypothesized to play a role in both the pathophysiology of migraines and the related cardiovascular risk^[1]^. Accordingly, we investigated the relationships between coronary vascular dysfunction and migraine history in a new cohort of women with signs and symptoms of ischemia and no obstructive coronary arteries (INOCA).

## METHODS

In the WISE-Coronary Vascular Dysfunction (WISE-CVD) project, 437 women with suspected INOCA completed standardized medical history and symptom questionnaires, including the Seattle Angina Questionnaire. Typical angina was defined as meeting all three criteria: (1) substernal discomfort; (2) precipitated by physical exertion or emotional stress; and (3) relieved within 10 min by rest or nitroglycerin. Nontypical angina was defined as all others not meeting the criteria for typical angina. Of the 279 women who underwent invasive coronary function testing, information pertaining to migraine headache was available for 252 women. Invasive coronary function testing was performed with a Doppler wire in the left anterior descending artery as the preferred artery, as previously described^[3]^. Coronary flow reserve (CFR) was calculated as the ratio of the average peak velocity (APV) at hyperemia to the APV at rest, in response to intracoronary adenosine infusions (18 and 36 mcg); we previously showed that this velocity reserve correlated closely with volumetric flow, and thus it was used to represent CFR^[4]^. Reduced microvascular function was defined as CFR ≤ 2.5. Endothelial-dependent function and the presence of epicardial spasm were evaluated in response to intracoronary infusion of acetylcholine (10^−4^ mol/L) at 18.2 mcg/mL for 3 min, at an infusion rate of 0.8 mL/min. Coronary blood flow (CBF) response to acetylcholine was calculated from the Doppler-obtained APV and vessel diameter at baseline and post-acetylcholine, using the following equation: CBF = π (APV/2) (vessel diameter/2)^2^. Failure to increase the coronary blood flow (ΔCBF) > 50% was defined as abnormal microvascular endothelial-dependent function. A failure of coronary artery diameter to increase (or the presence of vasospasm) was considered abnormal macrovascular endothelial-dependent function. Abnormal nonendothelial-dependent macrovascular function was defined as coronary diameter response ≤ 20% increase to intracoronary nitroglycerin 200 mcg. Wilcoxon rank sum test, *t*-test, and linear regression models were performed to compare those with and without migraine history.

## RESULTS

Table 1 compares the angina and coronary function testing results in the women stratified by migraine history. Migraine history was reported in 126 (50%) women. Compared to women without migraines, those with migraines were younger (52 ± 11 years *vs.* 56 ± 12 years, *P* = 0.006) and less frequently postmenopausal (60% *vs.* 76%, *P* = 0.01). There were no significant differences in body mass index or traditional cardiovascular risk factors comparing the two groups. While there was no difference in prevalence of typical *vs.* nontypical angina between the two groups, women with migraines had more frequent angina and poorer quality of life, which remained significant after adjusting for age. Women with migraines were also more likely to have angina with sexual activity, at rest, with strong emotions, with hot/cold temperatures, and waking them from sleep.

Compared to women without migraines, women with migraines had greater CBF response, suggesting better microvascular endothelial function, and they were less likely to have at least one abnormal coronary function measure. However, there was no difference in prevalence of coronary vascular dysfunction comparing the two groups, and linear regression models demonstrated no significant age-adjusted differences in the coronary function measures.

## DISCUSSION

Among women with suspected INOCA, migraine history was prevalent in half of the cohort, and those with migraines were younger and had worse angina compared to women without migraines. However, coronary vascular dysfunction by invasive coronary function testing was not associated with migraine history when adjusted by age. To our knowledge, these findings are the first to assess nonendothelial and endothelial-dependent coronary vascular function in women with and without reported migraine history.

The higher prevalence of angina in women with migraine history is consistent with a prior report from the Atherosclerosis Risk in Communities Study, which found that, among 12,409 men and women, those with a history of migraines and other headaches were more likely to have a history of angina than those without headaches^[5]^. While our angina data are consistent with the original WISE report of no difference in prevalence of typical or nontypical angina symptoms^[6]^, our detailed symptom questionnaires now highlight differences in angina triggers and other angina characteristics. We suspect this difference is likely related to the inclusion of women with both obstructive and nonobstructive coronary disease in the original WISE cohort, in which about a third had obstructive disease. This is in contrast to the WISE-CVD cohort, which included only women with no obstructive coronary arteries, allowing more specific analysis of angina suspected to be INOCA.

In our investigation, coronary vascular dysfunction as diagnosed by invasive coronary function testing was not significantly different in women with *vs.* without migraine history. The difference in coronary microvascular endothelial function (ΔCBF) was no longer significant when adjusted for age. Compared to women without migraine history, women with migraine history had a higher prevalence of normal invasive coronary function testing (18% *vs.* 6%, *P* = 0.01), which suggests that there may be alternate mechanisms contributing to their chest pain, such as heightened cardiac nociception or autonomic dysfunction^[7]^. Prior studies have suggested autonomic imbalance in patients with migraines, particularly in those with aura^[8]^. Indeed, we found that women with migraines had a higher prevalence of angina triggered by emotional stress and temperature extremes, which can also be triggers for migraine attacks^[9,10]^.

Invasive coronary function testing of this cohort did not include administration of high-dose acetylcholine (100 mcg) or 12-lead electrocardiographic monitoring in the assessment of epicardial and microvascular coronary spasm, thus limiting our ability to evaluate relationships between migraine history and vasospasm. A recent large study of patients with suspected INOCA undergoing invasive acetylcholine testing for coronary vasospasm found similar results with no significant difference in frequency of migraine in patients with epicardial vasospasm, microvascular vasospasm, and normal acetylcholine testing, although this study included both male and female patients, which may account for the lower frequency of migraines reported (11%)^[11]^. Since epicardial vasospasm has been found to be more prevalent in women than men with suspected INOCA^[12]^ and since women are three times more likely than men to have migraines^[13]^, further study of sex differences in vascular dysfunction and migraine may help understand the mechanisms related to increased cardiovascular risk in women with migraines.

Limitations to this current analysis include absence of available vascular or hormonal biomarkers that may help provide further insight, since migraine history was more frequently reported in premenopausal women. Additionally, the left anterior descending artery was the preferred artery for coronary function testing, thus the presence of coronary vascular dysfunction in other arteries was not evaluated. Another limitation of our cohort study pertains to self-reported migraine diagnosis rather than determined using International Headache Society criteria. Data regarding intake of specific migraine medication were not available to assess any potential relationships among antimigraine medication use, angina characteristics, and coronary vascular function, as ergotamine derivatives and triptans have coronary vasoconstrictive properties^[14]^.

In conclusion, our findings suggest that coronary vascular dysfunction may not be linked with migraine headaches to increase risk for cardiovascular events in women. Although our analysis did not demonstrate an association between migraine history and coronary vascular dysfunction, further larger, prospective studies that include higher dose acetylcholine-mediated vasospasm testing are needed to better understand the association between migraines and adverse cardiovascular outcomes in women.

## Figures and Tables

**Table 1.** Angina questionnaire and coronary function testing results in women stratified by migraine history

Results (mean ± SD or %)	No migraine history (*n* = 126)	Migraine history (*n* = 126)	*P* value
**Seattle Angina Questionnaire**			
Physical limitation	68 ± 25	65 ± 24	0.29
Angina stability	49 ± 26	44 ± 29	0.14
Angina frequency	68 ± 25	58 ± 26	**0.001**
Treatment satisfaction	73 ± 24	68 ± 24	0.19
Quality of life	54 ± 24	45 ± 23	**0.006**
SAQ-7 score	82 ± 22	73 ± 21	**0.002**
**Characteristics of angina**			
Typical angina	43 (37%)	55 (45%)	0.23
Non typical angina	73 (63%)	68 (55%)	
**What brings on these sensations?**			
Whole body exertion, *n* = 237	52 (44%)	60 (50%)	0.33
Sexual activity, *n* = 231	13 (12%)	30 (25%)	**0.007**
Strong emotions or stressful situations, *n* = 236	62 (53%)	78 (66%)	**0.03**
When it’s very hot or very cold, *n* = 237	28 (24%)	43 (36%)	**0.03**
Nothing at all (it happens at rest), *n* = 236	67 (57%)	86 (73%)	**0.01**
**What usually relieves these sensations?**			
Stopping activity that brought it on, *n* = 234	60 (52%)	63 (53%)	0.80
Rest, *n* = 235	70 (60%)	79 (66%)	0.34
Nitroglycerin, *n* = 235	37 (32%)	48 (40%)	0.18
Nothing seems to relieve it, *n* = 234	24 (21%)	20 (17%)	0.50
**Do these feelings ever**			
Wake you from sleep, *n* = 234	47 (40%)	81 (69%)	**< 0.0001**
**Coronary function measures**			
CFR to adenosine	2.7 ± 0.7	2.70 ± 0.56	0.99
ΔCBF to acetylcholine	56 ± 85	75 ± 84	**0.04**
Coronary diameter response to acetylcholine	−0.37 ± 15	−0.01 ± 15	0.55
Coronary diameter response to nitroglycerin	14 ± 14	16 ± 14	0.41
**Prevalence of coronary vascular dysfunction**			
Nonendothelial microvascular dysfunction (CFR < 2.5 to adenosine), *n* = 236	47 (40%)	41 (35%)	0.50
Endothelial microvascular dysfunction (ΔCBF ≤ 50% to acetylcholine), *n* = 190	54 (60%)	48 (48%)	0.11
Endothelial macrovascular dysfunction (diameter response ≤ 0% to acetylcholine), *n* = 223	55 (50%)	52 (46%)	0.69
Nonendothelial macrovascular response (diameter response ≤ 20% to nitroglycerin), *n* = 222	75 (67%)	69 (63%)	0.57
Any abnormal measure above, *n* = 184	83 (94%)	79 (82%)	**0.01**

Seattle Angina Questionnaire (0–100, lower value indicates worse angina); SAQ-7 (shortened version of the SAQ including the physical limitation, angina frequency, and quality of life domains). ΔCBF: Coronary blood flow change; CFR: coronary flow reserve; SAQ: Seattle Angina Questionnaire.
